# Correction: Spatiotemporal dynamics of PIEZO1 localization controls keratinocyte migration during wound healing

**DOI:** 10.7554/eLife.79034

**Published:** 2022-04-01

**Authors:** Jesse R Holt, Wei-Zheng Zeng, Elizabeth L Evans, Seung-Hyun Woo, Shang Ma, Hamid Abuwarda, Meaghan Loud, Ardem Patapoutian, Medha M Pathak

**Keywords:** Mouse

Holt JR, Zeng W-Z, Evans EL, Woo S-H, Ma S, Abuwarda H, Loud M, Patapoutian A, Pathak MM. 2021. Spatiotemporal dynamics of PIEZO1 localization controls keratinocyte migration during wound healing . *eLife* **10**:e65415. doi: 10.7554/eLife.65415.

Published 27 September 2021

After publication, we became aware that there was an inadvertent error in the pixel-to-micron conversion factors used for the trajectories reported in Figure 2 and related figure supplements. We have redone the analysis using the appropriate conversion factors for Figure 2, Figure 2-figure supplement 1, Figure 2—figure supplement 2, Figure 2—figure supplement 3. Following this re-analysis, the results for *Piezo1* GoF cells are slightly changed (GoF trajectories are straighter than for Con_GoF_ cells as reported, but there is no difference in cell speed). For *Piezo1* cKO data, while the scaling of plots is changed, the results do not change. We have corrected Figure 2 and related supplements, the related source data files, and the relevant Results description as detailed below. The main findings and conclusions of the work do not change.

The article has been corrected accordingly.

Corrected Figure 2:

**Figure 1:**
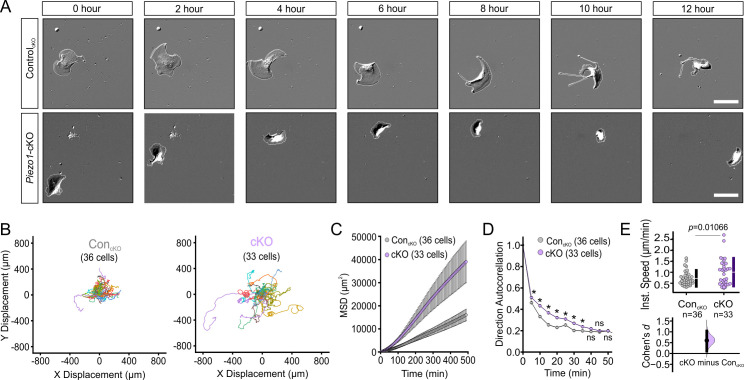


**PIEZO1 mediates speed and direction during single cell keratinocyte migration.** (**A**) Representative differential interference contrast (DIC) images from time-lapse series of individual migrating keratinocytes isolated from Control_cKO_ (*top*) and respective *Piezo1-*cKO mice (*bottom*). Thin white lines denote the cell boundary. Scale bar = 25 µm. (**B**) Cell trajectories derived from tracking single keratinocytes during time-lapse experiments. Trajectories are shown with cell position at time point 0 normalized to the origin. See also Figure 2—figure supplement 1. (**C**) Mean squared displacement (MSD) analysis of Control_cKO_ and *Piezo1-*cKO keratinocytes tracked in B. Average MSD is plotted as a function of time. Error bars (SEM) are smaller than symbols at some points. (**D**) Average direction autocorrelation measurement of *Piezo1-*cKO and Control_cKO_ keratinocytes plotted as a function of time interval. * denotes a statistically significant difference, and ns denotes ‘not statistically significant’. From left to right: *P* = 2.0307 × 10^–4^, 5.75675 × 10^–14^, 3.18447 × 10^–15^, 5.34662 × 10^–10^, 1.72352 × 10^–4^, 1.34648 × 10^–5^, 0.01951, 0.13381, 0.61758 as determined by Kruskal-Wallis test. Plotted error bars (SEM) are smaller than symbols. (**E**) Quantitation of the average instantaneous speed from individual *Piezo1-*cKO keratinocytes relative to control cells are shown in a Cumming plot (Cohen’s *d* = 0.6; *p* value calculated via Kolmogorov-Smirnov test). n in B–E denotes the number of individually migrating cells tracked. See also Figure 2—figure supplements 2–3 and Figure 2 videos 1 and 2. Data are from three independent experiments from two litters. Bars in upper Cumming plots denote mean ±  s.d.

Original Figure 2:

**Figure 2:**
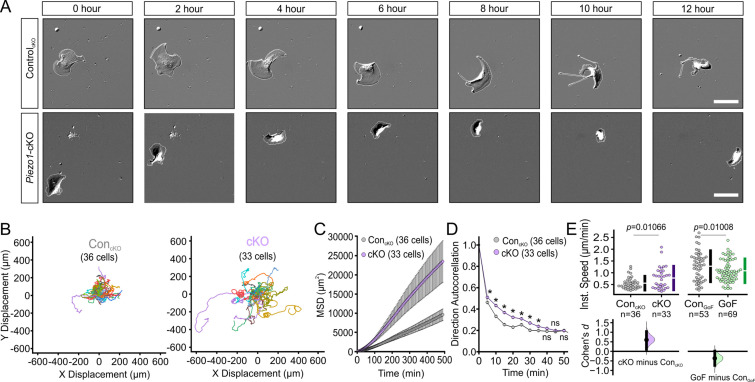


**PIEZO1 mediates speed and direction during single cell keratinocyte migration.** (**A**) Representative differential interference contrast (DIC) images from time-lapse series of individual migrating keratinocytes isolated from Control_cKO_ (*top*) and respective *Piezo1-*cKO mice (*bottom*). Thin white lines denote the cell boundary. Scale bar = 25 µm. (**B**) Cell trajectories derived from tracking single keratinocytes during time-lapse experiments. Trajectories are shown with cell position at time point 0 normalized to the origin. See also Figure 2—figure supplement 1. (**C**) Mean squared displacement (MSD) analysis of Control_cKO_ and *Piezo1-*cKO keratinocytes tracked in B. Average MSD is plotted as a function of time. Error bars (SEM) are smaller than symbols at some points. (**D**) Average direction autocorrelation measurement of *Piezo1-cKO* and Control_cKO_ keratinocytes plotted as a function of time interval. * denotes a statistically significant difference, and ns denotes ‘not statistically significant’. From left to right: *P* = 2.0307 × 10^–4^, 5.75675 × 10^–14^, 3.18447 × 10^–15^, 5.34662 × 10^–10^, 1.72352 × 10^–4^, 1.34648 × 10^–5^, 0.01951, 0.13381, 0.61758 as determined by Kruskal-Wallis test. Plotted error bars (SEM) are smaller than symbols. (**E**) Quantitation of the average instantaneous speed from individual *Piezo1-*cKO keratinocytes (*left*) and *Piezo1*-GoF keratinocytes (right) relative to the respective control cells are shown in a Cumming plot (Cohen’s *d* = 0.6 [*Piezo1-*cKO]; *d* = −0.362 [*Piezo1-*GoF]; *p* values calculated via Kolmogorov-Smirnov test). n in B–E denotes the number of individually migrating cells tracked. See also Figure 2—figure supplements 2–3 and Figure 2 videos 1 and 2. Data are from three independent experiments from two litters for conditional knockout (cKO) and six independent experiments from five litters for gain-of-function (GoF). Bars in upper Cumming plots denote mean ±  s.d.

**Corrected Results section:**

We observed that *Piezo1-*GoF keratinocytes also explored a somewhat larger area compared to littermate Con_GoF_ cells, due to the cells migrating straighter with no difference in cell speed (Figure 2- figure supplement 3). Overall, our data demonstrate that PIEZO1 regulates keratinocyte migration, with channel knockout resulting in faster migration speed. The effects of the GoF mutation were more complex, and both *Piezo1* knockout and the GoF mutation resulted in straighter trajectories.

**Original version:**

We observed no difference in the MSD plots of *Piezo1*-GoF keratinocytes and littermate Control_GoF_ cells (Figure 2—figure supplement 3). However, separating the data into directionality and speed indicated that *Piezo1*-GoF cells moved straighter (Figure 2—figure supplement 3) and slower (Figure 2E). Overall, our data demonstrate that PIEZO1 regulates keratinocyte migration, with channel activity resulting in slower migration speed. The effects on directionality were more complex, with both PIEZO1 knockout and a GoF mutation resulting in straighter trajectories.

Corrected Figure 2—figure supplement 1:

**Figure 3:**
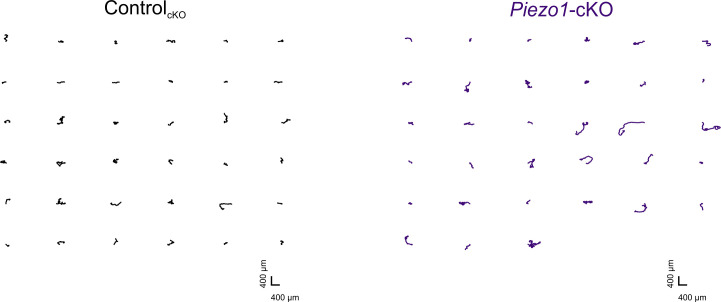


***Piezo1*-cKO keratinocytes migrate further**. Individual trajectories seen in Figure 2B from *Piezo1*-cKO (*right*) and Control_cKO_ (*left*) keratinocytes.

Original Figure 2–figure supplement 1:

**Figure 4:**
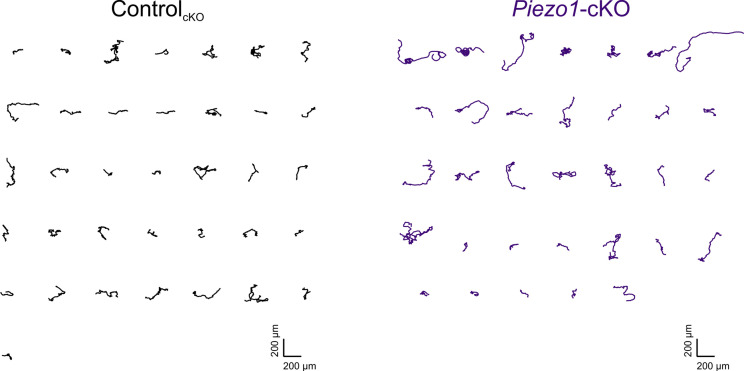


**Piezo1-cKO keratinocytes migrate further**. Individual trajectories seen in Figure 2B from Piezo1-cKO (right) and Control_cKO_ (left) keratinocytes.

Corrected Figure 2–figure supplement 2:

**Figure 5:**
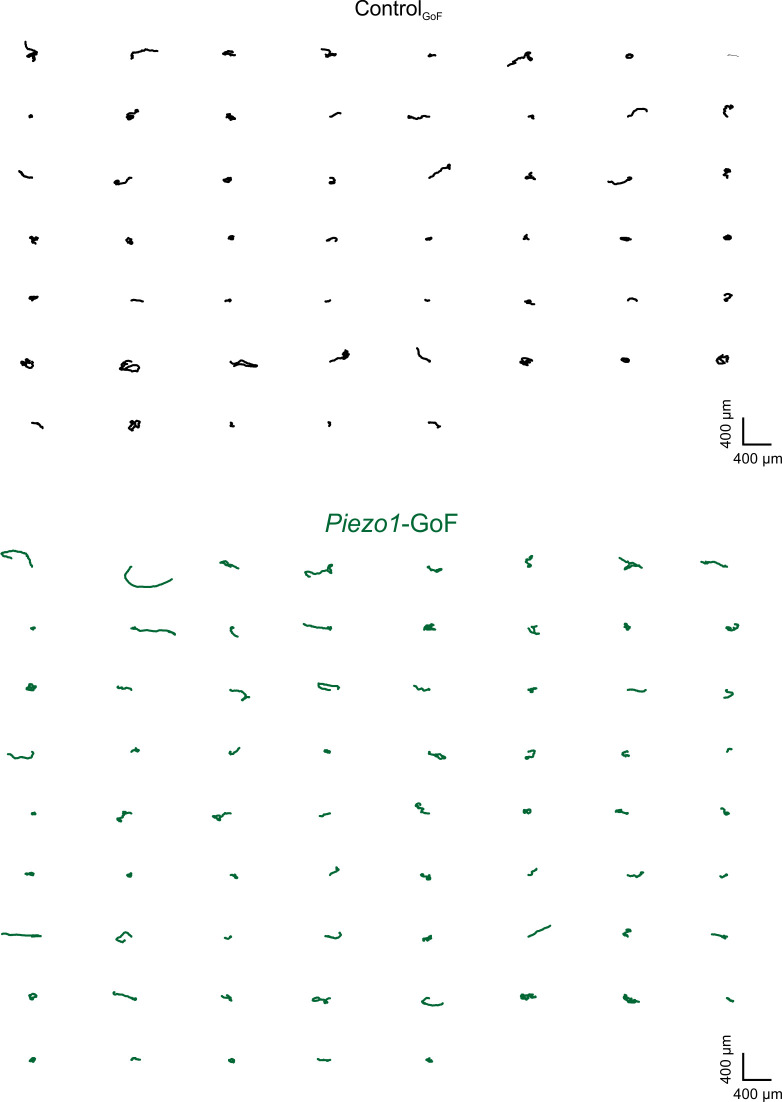


***Piezo1*-GoF keratinocytes migrate straighter**. Individual trajectories from *Piezo1*-GoF (*bottom*) and Control_GoF_ (*top*) keratinocytes.

Original Figure 2–figure supplement 2:

**Figure 6:**
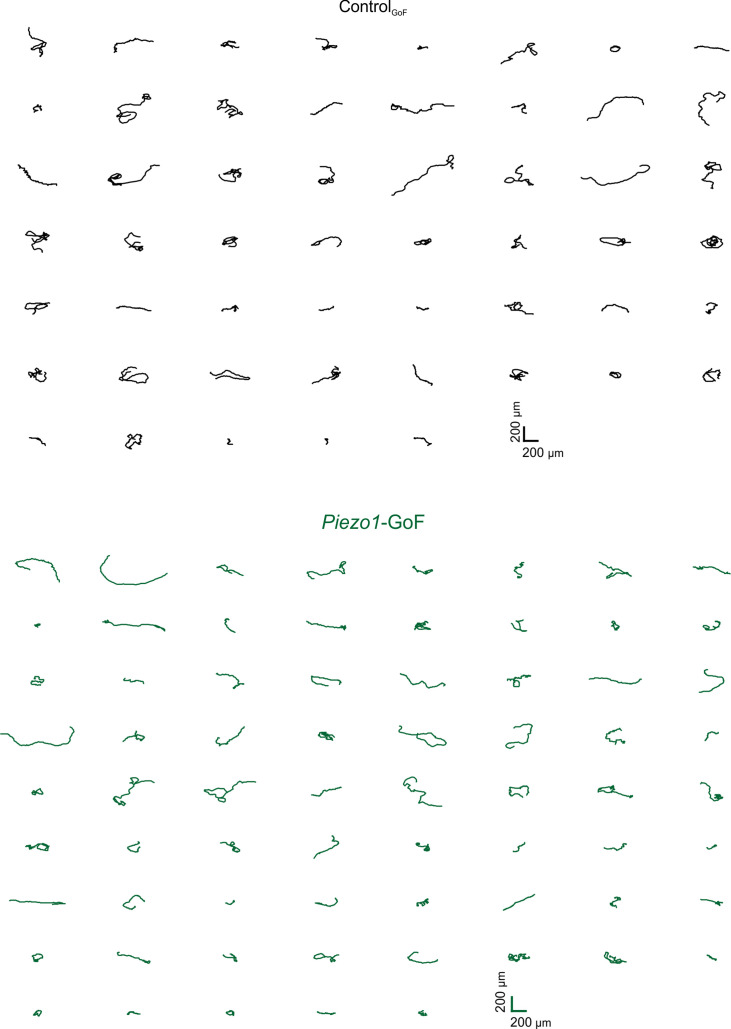


**Piezo1-GoF keratinocytes migrate straighter.** Individual trajectories from *Piezo1*-GoF (*bottom*) and Control_GoF_ (*top*) keratinocytes.

Corrected Figure 2–figure supplement 3

**Figure 7:**
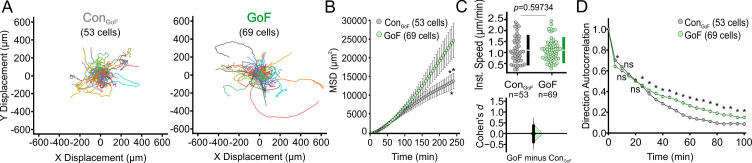


**Single cell migration of Piezo1-GoF keratinocytes.** (**A**) Cell trajectories derived from tracking Control_GoF_ (left) and *Piezo1-*GoF (right) keratinocytes during time-lapse experiments. Trajectories are shown with cell position at time point 0 normalized to the origin. (**B**) Mean squared displacement (MSD) analysis of Control_GoF_ and *Piezo1-*GoF keratinocytes tracked in A. Average MSD is plotted as a function of time. Error bars (SEM) are smaller than symbols at some points. From left to right, asterisks denote *P* = 0.04647, 0.04424, 0.03956 as determined by Kruskal-Wallis test. (**C**) Quantitation of the average instantaneous speed from individual *Piezo1*-GoF keratinocytes relative to control cells are shown in a Cumming plot (Cohen’s *d* = 0.0006; *p* value calculated via Kolmogorov-Smirnov test). (**D**) Average direction autocorrelation for *Piezo1-*GoF and Control_GoF_ keratinocytes plotted as a function of time interval. * denotes a statistically significant difference, and ns denotes ‘not statistically significant’. From left to right: *P* = 7.66613 × 10^–4^, 3.22610 × 10^–1^, 8.00140 × 10^–1^, 1.26680 × 10^–1^, 2.80100 × 10^–2^, 1.17235 × 10^–4^, 1.70078 × 10^–8^, 2.47432 × 10^–8^, 1.76724 × 10^–14^, 4.01667 × 10^–13^, 2.97488 × 10^–16^, 3.54287 × 10^–15^, 4.08085 × 10^–12^, 3.60513 × 10^–12^, 9.64395 × 10^–11^, 3.83225 × 10^–10^, 1.31866 × 10^–8^, 7.45673 × 10^–8^, 9.63184 × 10^–5^, and 4.16131 × 10^–5^ as determined by Kruskal-Wallis test. Related to Figure 2. Data are from six independent experiments from five litters. Data in B and D are presented as the mean ± SEM. See also Figure 2—video 2.

Original Figure 2–figure supplement 3

**Figure 8:**
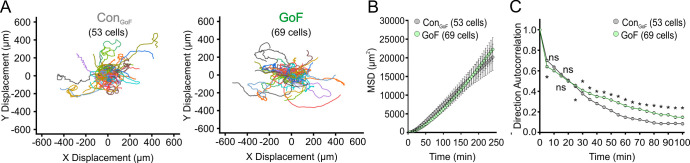


**Single cell migration of Piezo1-GoF keratinocytes.** (**A**) Cell trajectories derived from tracking Control_GoF_(left) and *Piezo1-*GoF (right) keratinocytes during time-lapse experiments. Trajectories are shown with cell position at time point 0 normalized to the origin. (**B**) Mean squared displacement (MSD) analysis of Control_GoF_ and *Piezo1-*GoF keratinocytes tracked in A. Average MSD is plotted as a function of time. Error bars (SEM) are smaller than symbols at some points. (**C**) Average direction autocorrelation for *Piezo1-*GoF and Control_GoF_ keratinocytes plotted as a function of time interval. * denotes a statistically significant difference, and ns denotes ‘not statistically significant’. From left to right: *P* = 7.922194 × 10^–4^, 0.3263, 0.8208, 0.14186, 0.02523, 1.03604 × 10^–4^, 1.42496 × 10^–8^, 2.18161 × 10^–8^, 1.71116 × 10^–14^, 3.13349 × 10^–13^, 3.0169 × 10^–16^, 2.17203 × 10^–15^, 3.3468 × 10^–12^, 2.83094 × 10^–12^, 1.17488 × 10^–10^, 3.54255 × 10^–10^, 1.21566 × 10^–8^, 7.6758 × 10^–8^, 8.58726 × 10^–5^, 3.76797 × 10^–5^ as determined by Kruskal-Wallis test. Related to Figure 2. Data are from six independent experiments from five litters. Data in B and C are presented as the mean ± SEM. See also Figure 2—video 2.

