# Supplementary material for: Spatiotemporal dynamics of PIEZO1 localization controls keratinocyte migration during wound healing
Source: eLife. 2021 Sep 27;10:e65415. doi: 10.7554/eLife.65415 (PMC8577841; doi:10.7554/eLife.65415)
Supplement: Figure 5—source code 1. — See also Code availability. [file elife-65415-fig5-code1.zip › 461627b6-518f-44f0-b3c0-2aaeb376a859.pdf]

2021_09_17_ADAPT_Analysis

September 18, 2021

[1]:
```python
from PIL import Image
import numpy as np
import pandas as pd
import openpyxl as xl
import matplotlib.pyplot as plt
from scipy.stats import t
import plotly.express as px
from glob import glob
import os
```

0.0.1 Define Image Paths for Velocity Maps to be read into Python

Define a function to take all the velocity heatmap outputs from ADAPT within a folder and then we create a for loop to go through the folder and write images to a dataframe to create heatmaps seen in Figure 5E and Supp Figure 9. The second for loop melts each heatmap into one column

[ ]:
```python
def load_data(path):
    images = sorted(glob(os.path.join(path,"Velocity Outputs", "*.tif")))
    return(images)

filepath="G:/Shared drives/Keratinocytes/04_Data/Data_Jesse/ADAPT"
filenames = load_data(filepath)

for file in filenames:
    taco = file
    print(taco)
    photo = Image.open(taco)
    filename = taco.split('/')[-1]
    print(filename)
    data= np.array(photo)
    df = pd.DataFrame(data)
    df_1 = df.replace(0, np.nan)
    df_1.to_excel('C:/Users/mrjes/Documents/Velocity Outputs/'+filename[:
 ↪-16]+'_VelocityMapswithNaN.xlsx')
```

[ ]:
```python
for file in filenames:
    taco = file
    print(taco)
```

```
    photo = Image.open(taco)
    filename = taco.split('/')[-1]
    print(filename)
    data=np.array(photo) #numpy array of the image
    df =pd.DataFrame(data) #puts it into a dataframe
    df_1 = df.replace(0, np.nan) #replace all 0's with NaN
    #df_1.dropna(inplace=True) #drop rows with NaN
    df_long = pd.melt(df_1) # put everything into one column
    df_long.to_csv('C:/Users/mrjes/Documents/Velocity Outputs/'+filename[:
 ↪-16]+'_VelocityMaps_Melted.csv') #ouputs to csv bc too large for excel writer
```

Load CSV files which have the heatmaps melted into one column and put them into one dataframe which contains every FOV from each condition melted into one column.

[ ]:

```
ConDMSO1 = pd.read_csv("C:/Users/mrjes/Documents/Velocity Outputs/
 ↪235_Control_A1_cp_masks_Output_VelocityMaps_Melted.csv")
ConDMSO2 = pd.read_csv("C:/Users/mrjes/Documents/Velocity Outputs/
 ↪2B_mKera_Predicted_Cell1_Output_VelocityMaps_Melted.csv")
ConDMSO3 = pd.read_csv("C:/Users/mrjes/Documents/Velocity Outputs/
 ↪2B_mKera_Predicted_Cell2_Output_VelocityMaps_Melted.csv")
ConDMSO4 = pd.read_csv("C:/Users/mrjes/Documents/Velocity Outputs/
 ↪2B_mKera_Predicted_Cell_3_VelocityMaps_Melted.csv")

all_ConDMSO =[ConDMSO1.loc[:, 'value'], ConDMSO2.loc[:, 'value'], ConDMSO3.loc[:
 ↪, 'value'], ConDMSO4.loc[:, 'value']]
all_ConDMSO_df = pd.concat(all_ConDMSO)
all_ConDMSO_df.to_csv('C:/Users/mrjes/Documents/Velocity Outputs/
 ↪Con+DMSO_Melted.csv') #ouputs to csv bc too large for excel writer
print('Con+DMSO to csv')

ConYoda1 = pd.read_csv("C:/Users/mrjes/Documents/Velocity Outputs/
 ↪235_Control_a_Y1-fillHoles_Output_VelocityMaps_Melted.csv")
ConYoda2 = pd.read_csv("C:/Users/mrjes/Documents/Velocity Outputs/
 ↪3B_Cpontrol+Yoda1-Cell2_Output_VelocityMaps_Melted.csv")
ConYoda3 = pd.read_csv("C:/Users/mrjes/Documents/Velocity Outputs/
 ↪3B_Control+Yoda1_Cell1_Output2_VelocityMaps_Melted.csv")
ConYoda4 = pd.read_csv("C:/Users/mrjes/Documents/Velocity Outputs/
 ↪3B_Control+Yoda1_Cell3_Output_VelocityMaps_Melted.csv")

all_ConY1 =[ConYoda1.loc[:, 'value'], ConYoda2.loc[:, 'value'], ConYoda3.loc[:,␣
 ↪'value'], ConYoda4.loc[:, 'value']]
all_ConY1_df = pd.concat(all_ConY1)
all_ConY1_df.to_csv('C:/Users/mrjes/Documents/Velocity Outputs/Con+Y1_Melted.
 ↪csv') #ouputs to csv bc too large for excel writer
print('Con+Y1 to csv')
```

```python
cKO1 = pd.read_csv("C:/Users/mrjes/Documents/Velocity Outputs/
↪237_cKO_A3Output1_VelocityMaps_Melted.csv")
cKO2 = pd.read_csv("C:/Users/mrjes/Documents/Velocity Outputs/
↪237_cKO_A3Output_VelocityMaps_Melted.csv")
cKO3 = pd.read_csv("C:/Users/mrjes/Documents/Velocity Outputs/
↪235_cKO_Output2_VelocityMaps_Melted.csv")
cKO4 = pd.read_csv("C:/Users/mrjes/Documents/Velocity Outputs/
↪235_cKO_Output_VelocityMaps_Melted.csv")

all_cKO =[cKO1.loc[:, 'value'], cKO2.loc[:, 'value'], cKO3.loc[:, 'value'],␣
↪cKO4.loc[:, 'value']]
all_cKO_df = pd.concat(all_cKO)
all_cKO_df.to_csv('C:/Users/mrjes/Documents/Velocity Outputs/cKO_Melted.csv')␣
↪#ouputs to csv bc too large for excel writer
print('cKO to csv')

Con_G_Back1 = pd.read_csv("C:/Users/mrjes/Documents/Velocity Outputs/
↪284_GoF_Quad1_Output0_VelocityMaps_Melted.csv")
Con_G_Back2 = pd.read_csv("C:/Users/mrjes/Documents/Velocity Outputs/
↪284_GoF_Quad1_Output1_VelocityMaps_Melted.csv")
Con_G_Back3 = pd.read_csv("C:/Users/mrjes/Documents/Velocity Outputs/
↪349_2021_07_13_Batch73_Skin8_4_Cell0_VelocityMaps_Melted.csv")
Con_G_Back4 = pd.read_csv("C:/Users/mrjes/Documents/Velocity Outputs/
↪349_2021_07_13_Batch73_Skin8_4_Cell1_VelocityMaps_Melted.csv")
Con_G_Back5 = pd.read_csv("C:/Users/mrjes/Documents/Velocity Outputs/
↪349_2021_07_13_Batch73_Skin8_6_VelocityMaps_Melted.csv")

all_Con_GofBackground =[Con_G_Back1.loc[:, 'value'], Con_G_Back2.loc[:,␣
↪'value'], Con_G_Back3.loc[:, 'value'],  Con_G_Back4.loc[:, 'value'], ␣
↪Con_G_Back5.loc[:, 'value']]
all_Con_GofBackground_df = pd.concat(all_Con_GofBackground)
all_Con_GofBackground_df.to_csv('C:/Users/mrjes/Documents/Velocity Outputs/
↪All_Con_GoFBackground_Melted.csv') #ouputs to csv bc too large for excel␣
↪writer
print('Control (GoF Background) written to csv')

GoF_1 = pd.read_csv("C:/Users/mrjes/Documents/Velocity Outputs/
↪284_GoF_Pos2_VelocityMaps_Melted.csv")
GoF_2 = pd.read_csv("C:/Users/mrjes/Documents/Velocity Outputs/
↪349_Batch73_Skin6-3_VelocityMaps_Melted.csv")
GoF_3 = pd.read_csv("C:/Users/mrjes/Documents/Velocity Outputs/
↪349_Batch73_Skin6-3_Cell1_VelocityMaps_Melted.csv")
GoF_4 = pd.read_csv("C:/Users/mrjes/Documents/Velocity Outputs/
↪349_2021_07_17_Batch73_Skin6_1_VelocityMaps_Melted.csv")
GoF_5 = pd.read_csv("C:/Users/mrjes/Documents/Velocity Outputs/
↪349_2021_07_13_Batch73_Skin6_2_VelocityMaps_Melted.csv")
```

```
GoF_6 = pd.read_csv("C:/Users/mrjes/Documents/Velocity Outputs/
↪284_GoF_Pos3_VelocityMaps_Melted.csv")

all_GoF =[GoF_1.loc[:, 'value'], GoF_2.loc[:, 'value'], GoF_3.loc[:, 'value'],␣
↪GoF_4.loc[:, 'value'], GoF_5.loc[:, 'value'], GoF_6.loc[:, 'value']]
all_GoF_df = pd.concat(all_GoF)
all_GoF_df.to_csv('C:/Users/mrjes/Documents/Velocity Outputs/All_GoF_Melted.
↪csv') #ouputs to csv bc too large for excel writer
print('GoF Written to csv')
```

0.0.2 Figure 5D statistics:

Now that we have all of our velocity maps loaded into python as matrixes (cell 2) and the matrices were melted down into one column (cell 3) and saved into csv's so that we are looking at the velocity at every point alonng the cell boundary at every point for each condition (cell 4). We will load the CSV files which have those melted matrixes and then be able to apply statistics in Pyhon to these Dataframes

[ ]:
```
from numpy import mean
from numpy import std
from numpy import var
from math import sqrt
import dabest
from scipy import stats
import scipy
import pandas as pd
import dask.dataframe as dd
import seaborn
from dask.diagnostics import ProgressBar
import matplotlib.pyplot as plt
```

[ ]:
```
#load csv's which contain velocities from each condition in one column
all_ConDMSO_df = pd.read_csv('C:/Users/17605/Downloads/Con+DMSO_Melted.csv')
all_ConY1_df = pd.read_csv('C:/Users/17605/Downloads/Con+Y1_Melted.csv')
all_cKO_df=  pd.read_csv('C:/Users/17605/Downloads/cKO_Melted.csv')
all_Con_GofBackground_df=  pd.read_csv('C:/Users/17605/Downloads/
↪All_Con_GoFBackground_Melted.csv')
all_GoF_df=  pd.read_csv('C:/Users/17605/Downloads/All_GoF_Melted.csv')
```

[ ]:
```
all_ConDMSO_df = all_ConDMSO_df.loc[:, 'value']
all_cKO_df = all_cKO_df.loc[:, 'value']
all_ConY1_df = all_ConY1_df.loc[:, 'value']
all_GoF_df = all_GoF_df.loc[:, 'value']
all_Con_GofBackground_df = all_Con_GofBackground_df.loc[:, 'value']

all_ConDMSO_df.dropna(inplace=True)
all_cKO_df.dropna(inplace=True)
```

```python
all_ConY1_df.dropna(inplace=True)
all_GoF_df.dropna(inplace=True)
all_Con_GofBackground_df.dropna(inplace=True)

abs_ConDMSO = abs(all_ConDMSO_df)
abs_cKODMSO = abs(all_cKO_df)
abs_ConY1 = abs(all_ConY1_df)
abs_Con_GofBackground = abs(all_Con_GofBackground_df)
abs_GoF = abs(all_GoF_df)

abs_ConDMSO.dropna(inplace=True)
abs_cKODMSO.dropna(inplace=True)
abs_ConY1.dropna(inplace=True)
abs_Con_GofBackground.dropna(inplace=True)
abs_GoF.dropna(inplace=True)

#create dataframes where we only have positive or negative numbers and check that they add to the total dataframe length
neg_ConDMSO = all_ConDMSO_df.to_frame().loc[(all_ConDMSO_df<0)]
pos_ConDMSO = all_ConDMSO_df.to_frame().loc[(all_ConDMSO_df>0)]
print("\nDoes Negative+Positive = length of total Con+DMSO?",len(neg_ConDMSO)+len(pos_ConDMSO)==len(all_ConDMSO_df))
neg_cKO = all_cKO_df.to_frame().loc[(all_cKO_df<0)]
pos_cKO = all_cKO_df.to_frame().loc[(all_cKO_df>0)]
print("\nDoes Negative+Positive = length of total cKO?",len(neg_cKO)+len(pos_cKO)==len(all_cKO_df))
neg_ConY1 = all_ConY1_df.to_frame().loc[(all_ConY1_df<0)]
pos_ConY1 = all_ConY1_df.to_frame().loc[(all_ConY1_df>0)]
print("\nDoes Negative+Positive = length of total Yoda1?",len(neg_ConY1)+len(pos_ConY1)==len(all_ConY1_df))
neg_GoF = all_GoF_df.to_frame().loc[(all_GoF_df<0)]
pos_GoF = all_GoF_df.to_frame().loc[(all_GoF_df>0)]
print("\nDoes Negative+Positive = length of total GoF?",len(neg_GoF)+len(pos_GoF)==len(all_GoF_df))
neg_Con_GoFBK = all_Con_GofBackground_df.to_frame().loc[(all_Con_GofBackground_df<0)]
pos_Con_GoFBK = all_Con_GofBackground_df.to_frame().loc[(all_Con_GofBackground_df>0)]
print("\nDoes Negative+Positive = length of total Control(GoF)?",len(neg_Con_GoFBK)+len(pos_Con_GoFBK)==len(all_Con_GofBackground_df))

abs_neg_ConDMSO = abs(neg_ConDMSO)
abs_neg_cKO = abs(neg_cKO)
abs_neg_ConY1 = abs(neg_ConY1)
abs_neg_GoF = abs(neg_GoF)
abs_neg_Con_GoFBK = abs(neg_Con_GoFBK)
```

```
abs_pos_ConDMSO = abs(pos_ConDMSO)
abs_pos_cKO = abs(pos_cKO)
abs_pos_ConY1 = abs(pos_ConY1)
abs_pos_GoF = abs(pos_GoF)
abs_pos_Con_GoFBK = abs(pos_Con_GoFBK)
```

[ ]:
```
#Combined Dataframe of test groups
Con_v_cKO_DF = pd.concat([all_ConDMSO_df.to_frame(), all_cKO_df.to_frame()],␣
 ↪axis=1)
Con_v_cKO_DF.columns.values[0] ='Con'
Con_v_cKO_DF.columns.values[1] ='cKO'
neg_Con_v_cKO_DF = pd.concat([neg_ConDMSO, neg_cKO], axis=1, ignore_index=True,␣
 ↪names= ['Con(Negative Velocities)','cKO(Negative Velocities)'] )
pos_Con_v_cKO_DF = pd.concat([pos_ConDMSO, pos_cKO], axis=1, ignore_index=True,␣
 ↪names= ['Con(Positive Velocities)','cKO(Positive Velocities)'])

Con_v_Y1_DF = pd.concat([all_ConDMSO_df.to_frame(), all_ConY1_df.to_frame()],␣
 ↪axis=1)
Con_v_Y1_DF.columns.values[0] ='Con+DMSO'
Con_v_Y1_DF.columns.values[1] ='Con+Y1'
neg_Con_v_Y1_DF = pd.concat([neg_ConDMSO, neg_ConY1], axis=1,␣
 ↪ignore_index=True, names= ['Con+DMSO(Negative Velocities)','Con+Y1(Negative␣
 ↪Velocities)'])
pos_Con_v_Y1_DF = pd.concat([pos_ConDMSO, pos_ConY1], axis=1,␣
 ↪ignore_index=True, names= ['Con+DMSO(Positive Velocities)','Con+Y1(Positive␣
 ↪Velocities)'])

Con_v_GoF_DF = pd.concat([all_Con_GofBackground_df.to_frame(), all_GoF_df.
 ↪to_frame()], axis=1)
Con_v_GoF_DF.columns.values[0] ='Control'
Con_v_GoF_DF.columns.values[1] ='GoF'
neg_Con_v_GoF_DF = pd.concat([neg_Con_GoFBK, neg_GoF], axis=1, names=␣
 ↪['Con(GoF)(Negative Velocities)','GoF(Negative Velocities)'])
pos_Con_v_GoF_DF = pd.concat([pos_Con_GoFBK, pos_GoF], axis=1, names=␣
 ↪['Con(GoF)(Positive Velocities)','GoF(Positive Velocities)'])

all_conditions_DF = pd.concat([Con_v_cKO_DF, Con_v_Y1_DF, Con_v_GoF_DF], axis=1)
all_conditions_neg_DF = pd.concat([neg_Con_v_cKO_DF, neg_Con_v_Y1_DF,␣
 ↪neg_Con_v_GoF_DF], axis=1, ignore_index=True)
all_conditions_pos_DF = pd.concat([pos_Con_v_cKO_DF, pos_Con_v_Y1_DF,␣
 ↪pos_Con_v_GoF_DF], axis=1, ignore_index=True)
```

0.0.3 Descriptive Statistics of the datasets

This code block will show the the normlity test results, MW p values, means, range, anddataset size for each condition. The following block will also show the Cohens d value for effect size.

[ ]:

```
Con_mean_vel = mean(all_ConDMSO_df)
Con_size= len(all_ConDMSO_df)
Con_range = (all_ConDMSO_df.min(),all_ConDMSO_df.max())
print("Con+DMSO normality test results:")
k2, p = stats.normaltest(all_ConDMSO_df)
alpha = 1e-3
print("p = {:g}".format(p))
if p < alpha:  # null hypothesis: x comes from a normal distribution
    print("The null hypothesis can be rejected")
else:
    print("The null hypothesis cannot be rejected")


allCKO_mean_vel = mean(all_cKO_df)
cKO_size= len(all_cKO_df)
cKO_range = (all_cKO_df.min(), all_cKO_df.max())
print("cKO background normality test results:")
k2, p = stats.normaltest(all_cKO_df)
alpha = 1e-3
print("p = {:g}".format(p))
if p < alpha:  # null hypothesis: x comes from a normal distribution
    print("The null hypothesis can be rejected")
else:
    print("The null hypothesis cannot be rejected")

scipy.stats.mannwhitneyu(all_ConDMSO_df, all_cKO_df)

ConY1_size= len(all_ConY1_df)
ConY1_mean_vel = mean(all_ConY1_df)
ConY1_range = (all_ConY1_df.min(), all_ConY1_df.max())
print("Con+Yoda1 normality test results:")
k2, p = stats.normaltest(all_ConY1_df)
alpha = 1e-3
print("p = {:g}".format(p))
if p < alpha:  # null hypothesis: x comes from a normal distribution
    print("The null hypothesis can be rejected")
else:
    print("The null hypothesis cannot be rejected")

GoF_size= len(all_GoF_df)
GoF_mean_vel = mean(all_GoF_df)
GoF_range = (all_GoF_df.min(), all_GoF_df.max())
print("GoF normality test results:")
k2, p = stats.normaltest(all_GoF_df)
alpha = 1e-3
print("p = {:g}".format(p))
if p < alpha:  # null hypothesis: x comes from a normal distribution
```

```
    print("The null hypothesis can be rejected")
else:
    print("The null hypothesis cannot be rejected")

Con_GoFBK_size= len(all_Con_GofBackground_df)
Con_GofBkg_vel = mean(all_Con_GofBackground_df)
Con_GofBkg_range = (all_Con_GofBackground_df.min(), all_Con_GofBackground_df.
 ↪max())
print("Control (GoF Background) normality test results:")
k2, p = stats.normaltest(all_Con_GofBackground_df)
alpha = 1e-3
print("p = {:g}".format(p))
if p < alpha:  # null hypothesis: x comes from a normal distribution
    print("The null hypothesis can be rejected")
else:
    print("The null hypothesis cannot be rejected")


(all_Con_v_cKO_stats, all_Con_v_cKO_p) = stats.mannwhitneyu(all_ConDMSO_df,␣
 ↪all_cKO_df)
(neg_Con_v_cKO_stats, neg_Con_v_cKO_p) = stats.mannwhitneyu(neg_ConDMSO,␣
 ↪neg_cKO)
(pos_Con_v_cKO_stats, pos_Con_v_cKO_p) = stats.mannwhitneyu(pos_ConDMSO,␣
 ↪pos_cKO)
(abs_Con_v_cKO_stats, abs_Con_v_cKO_p) = stats.mannwhitneyu(abs_ConDMSO,␣
 ↪abs_cKODMSO)

(all_Con_v_Y1_stats, all_Con_v_Y1_p) = stats.mannwhitneyu(all_ConDMSO_df,␣
 ↪all_ConY1_df)
(neg_Con_v_Y1_stats, neg_Con_v_Y1_p) = stats.mannwhitneyu(neg_ConDMSO,␣
 ↪neg_ConY1)
(pos_Con_v_Y1_stats, pos_Con_v_Y1_p) = stats.mannwhitneyu(pos_ConDMSO,␣
 ↪pos_ConY1)
(abs_Con_v_Y1_stats, abs_Con_v_Y1_p) = stats.mannwhitneyu(abs_ConDMSO,␣
 ↪abs_ConY1)

(all_Con_v_GoF_stats, all_Con_v_GoF_p) = stats.
 ↪mannwhitneyu(all_Con_GofBackground_df, all_GoF_df)
(neg_Con_v_GoF_stats, neg_Con_v_GoF_p) = stats.mannwhitneyu(neg_Con_GoFBK,␣
 ↪neg_GoF)
(pos_Con_v_GoF_stats, pos_Con_v_GoF_p) = stats.mannwhitneyu(pos_Con_GoFBK,␣
 ↪pos_GoF)
(abs_Con_v_GoF_stats, abs_Con_v_GoF_p) = stats.
 ↪mannwhitneyu(abs_Con_GofBackground, abs_GoF)

print("\nCon v cKO (all) MW p value = ", all_Con_v_cKO_p)
```

```
print("Con v cKO (neg) MW p value =", neg_Con_v_cKO_p)
print("Con v cKO (pos) MW p value = ", pos_Con_v_cKO_p)
print("Con v cKO (abs) MW p value = ", abs_Con_v_cKO_p)
print("\nCon v +Y1 (all) MW p value = ", all_Con_v_Y1_p)
print("Con v +Y1 (neg) MW p value = ", neg_Con_v_Y1_p)
print("Con v +Y1 (pos) MW p value = ", pos_Con_v_Y1_p)
print("Con v +Y1 (abs) MW p value = ", abs_Con_v_Y1_p)
print("\nCon v GoF (all) MW p value = ", all_Con_v_GoF_p)
print("Con v GoF (neg) MW p value = ", neg_Con_v_GoF_p)
print("Con v GoF (pos) MW p value = ", pos_Con_v_GoF_p)
print("Con v GoF (abs) MW p value = ", abs_Con_v_GoF_p)

print("\nCon+DMSO Dataset size = {:g}".format(Con_size))
print("cKO+DMSO Dataset size = {:g}".format(cKO_size))
print("Con+Y1 Dataset size = {:g}".format(ConY1_size))
print("GoF Dataset size = {:g}".format(GoF_size))
print("Control (GoF Background) Dataset size = {:g}".format(Con_GoFBK_size))

print("\nCon+DMSO Mean Velocity = {:g}".format(Con_mean_vel))
print("cKO+DMSO Mean Velocity = {:g}".format(allCKO_mean_vel))
print("Con+Yoda1 Mean Velocity = {:g}".format(ConY1_mean_vel))
print("GoF Mean Velocity = {:g}".format(GoF_mean_vel))
print("Control (GoF Bkgnd) Mean Velocity = {:g}".format(Con_GofBkg_vel))

print("\nNegative Con+DMSO Mean Velocity =", mean(neg_ConDMSO))
print("Negative cKO+DMSO Mean Velocity =", mean(neg_cKO))
print("Negative Control+Y1 Mean Velocity =", mean(neg_ConY1))
print("Negative GoF Mean Velocity =", mean(neg_GoF))
print("Negative Control (GoF Bkgnd) Mean Velocity =", mean(neg_Con_GoFBK))

print("\nPositive Con+DMSO Mean Velocity =", mean(pos_ConDMSO))
print("Positive cKO+DMSO Mean Velocity =", mean(pos_cKO))
print("Positive Control+Y1 Mean Velocity =", mean(pos_ConY1))
print("Positive GoF Mean Velocity =", mean(pos_GoF))
print("Positive Control (GoF Bkgnd) Mean Velocity =", mean(pos_Con_GoFBK))

print("\nCon+DMSO Mean Velocity (Absolute Value) =", mean(abs_ConDMSO))
print("cKO+DMSO Mean Velocity (Absolute Value) =", mean(abs_cKODMSO))
print("Con+Y1 Mean Velocity (Absolute Value) =", mean(abs_ConY1))
print("GoF Mean Velocity (Absolute Value) =", mean(abs_GoF))
print("Control (GoF Bkgnd) Mean Velocity (Absolute Value) =",␣
↪mean(abs_Con_GofBackground))

print("\nControl+DMSO Velocity range =", Con_range)
print("cKO Velocity range =", cKO_range)
print("Con+Y1 Velocity range =",ConY1_range)
print("GoF Velocity range =", GoF_range)
```

```python
print("Control (GoF Bkg) Velocity range =", Con_GofBkg_range)

#define cohens d using the function defined here: https://
↪machinelearningmastery.com/effect-size-measures-in-python/
def cohend(d1, d2):
    # calculate the size of samples
    n1, n2 = len(d1), len(d2)
    # calculate the variance of the samples
    s1, s2 = var(d1, ddof=1), var(d2, ddof=1)
    # calculate the pooled standard deviation
    s = sqrt(((n1 - 1) * s1 + (n2 - 1) * s2) / (n1 + n2 - 2))
    # calculate the means of the samples
    u1, u2 = mean(d1), mean(d2)
    # calculate the effect size
    return (u2 - u1) / s

d = cohend(neg_ConDMSO, neg_ConY1)
print('\nNegative Velocity: Con+DMSO v +Y1 Cohens d: %.3f' % d)
d = cohend(pos_ConDMSO, pos_ConY1)
print('Positive Velocity: Con+DMSO v +Y1 Cohens d: %.3f' % d)
d = cohend(neg_ConDMSO, neg_cKO)
print('\nNegative Velocity: Con v cKO Cohens d: %.3f' % d)
d = cohend(pos_ConDMSO, pos_cKO)
print('Positive Velocity: Con v cKO Cohens d: %.3f' % d)
d = cohend(neg_Con_GoFBK, neg_GoF)
print('\nNegative Velocity: Con v GoF  Cohens d: %.3f' % d)
d = cohend(pos_Con_GoFBK, pos_GoF)
print('Positive Velocity: Con v GoF Cohens d: %.3f' % d)

d = cohend(abs_ConDMSO, abs_ConY1)
print('\nAbsolute Value of all Con v +Y1 Velocity Cohens d: %.3f' % d)
d = cohend(abs_ConDMSO, abs_cKODMSO)
print('Absolute Value of all Con v KO Velocity Cohens d: %.3f' % d)
d = cohend(abs_Con_GofBackground, abs_GoF)
print('Absolute Value of all Con v GoF Velocity Cohens d: %.3f' % d)

d = cohend(all_ConDMSO_df, all_cKO_df)
print('\nAll Values Con v cKO Velocity Cohens d: %.3f' % d)
d = cohend(all_ConDMSO_df, all_ConY1_df)
print('All Values Con v +Y1 Velocity  Cohens d: %.3f' % d)
d = cohend(all_Con_GofBackground_df, all_GoF_df)
print('All Values Con v GoF Velocity Cohens d: %.3f' % d)
```

0.0.4 Output data to csv for creating figure 5D in Originlab

[ ]:
```
all_conditions_DF = pd.concat([neg_ConDMSO,neg_cKO, neg_ConY1,␣
↪neg_Con_GoFBK,neg_GoF, pos_ConDMSO,pos_cKO, pos_ConY1,␣
↪pos_GoF,pos_Con_GoFBK], axis=1)
all_conditions_DF.columns.values[0] ='Con+DMSO Negative Velocities'
all_conditions_DF.columns.values[1] ='cKO+DMSO Negative Velocities'
all_conditions_DF.columns.values[2] ='Con+Yoda1 Negative Velocities'
all_conditions_DF.columns.values[3] ='Con(Gof) Negative Velocities'
all_conditions_DF.columns.values[4] ='GoF Negative Velocities'
all_conditions_DF.columns.values[5] ='Con+DMSO Positive Velocities'
all_conditions_DF.columns.values[6] ='cKO+DMSO Positive Velocities'
all_conditions_DF.columns.values[7] ='Con+Yoda1 Positive Velocities'
all_conditions_DF.columns.values[8] ='Con(Gof) Positive Velocities'
all_conditions_DF.columns.values[9] ='GoF Positive Velocities'
all_conditions_DF.to_csv('C:/Users/17605/Downloads/2021_09_16_Fig5D_Velocities.
↪csv')
```

[ ]:
```
```
