# Supplementary material for: Spatiotemporal dynamics of PIEZO1 localization controls keratinocyte migration during wound healing
Source: eLife. 2021 Sep 27;10:e65415. doi: 10.7554/eLife.65415 (PMC8577841; doi:10.7554/eLife.65415)
Supplement: Transparent reporting form [file elife-65415-transrepform1.pdf]

Westbrook Centre, Milton Road
Cambridge CB4 1YG
UK

P 01223 855340
W elifesciences.org
T @elife

eLife's transparent reporting form

We encourage authors to provide detailed information *within their submission* to facilitate the interpretation and replication of experiments. Authors can upload supporting documentation to indicate the use of appropriate reporting guidelines for health-related research (see EQUATOR Network), life science research (see the BioSharing Information Resource), or the ARRIVE guidelines for reporting work involving animal research. Where applicable, authors should refer to any relevant reporting standards documents in this form.

If you have any questions, please consult our Journal Policies and/or contact us: editorial@elifesciences.org.

Sample-size estimation

- You should state whether an appropriate sample size was computed when the study was being designed
- You should state the statistical method of sample size computation and any required assumptions
- If no explicit power analysis was used, you should describe how you decided what sample (replicate) size (number) to use

Please outline where this information can be found within the submission (e.g., sections or figure legends), or explain why this information doesn't apply to your submission:

No statistical method was used to predetermine sample size. The sample size was chosen based on common practices in the field. Sample sizes and number of independent replicates for all experiments are included in relevant figure legends, and the Methods section.

Replicates

- You should report how often each experiment was performed
- You should include a definition of biological versus technical replication
- The data obtained should be provided and sufficient information should be provided to indicate the number of independent biological and/or technical replicates
- If you encountered any outliers, you should describe how these were handled
- Criteria for exclusion/inclusion of data should be clearly stated
- High-throughput sequence data should be uploaded before submission, with a private link for reviewers provided (these are available from both GEO and ArrayExpress)

Please outline where this information can be found within the submission (e.g., sections or figure legends), or explain why this information doesn't apply to your submission:

All experiments were independently reproduced as described in the Materials and Methods to reliably support the experimental findings stated in the manuscript. The biological replicate and experiment replication information are detailed in figure legends and Methods section. We define "biological replicates" as the number of mice for *in vivo* experiments, cells isolated from different pups or litters (in cases where cells from multiple pups of the same genotype were pooled), or cells independently treated with chemical agents.

1 data point was excluded from each of the Control (Piezo1-cKO background; out of 70 total data points), Control (Piezo1-GoF background; out of 40 total data points) (Fig. 1E) and Piezo1-GoF (out of 53 total data points) flicker datasets (Fig. 1H). We identified these data points as being outliers by the Grubb's test. Tracks detected by Kymobutler that were either (a) obvious noise from artifacts in the kymographs or (b) that were located away from the wound edge were removed from any subsequent analyses.

Statistical reporting

- Statistical analysis methods should be described and justified
- Raw data should be presented in figures whenever informative to do so (typically when N per group is less than 10)
- For each experiment, you should identify the statistical tests used, exact values of N, definitions of center, methods of multiple test correction, and dispersion and precision measures (e.g., mean, median, SD, SEM, confidence intervals; and, for the major substantive results, a measure of effect size (e.g., Pearson's r, Cohen's d)
- Report exact p-values wherever possible alongside the summary statistics and 95% confidence intervals. These should be reported for all key questions and not only when the p-value is less than 0.05.

Please outline where this information can be found within the submission (e.g., sections or figure legends), or explain why this information doesn't apply to your submission:

Statistical tests used, sample size, Cohen's *d*, *p*-values and all other relevant parameters are described in the corresponding figure legend, and Statistical Analysis section of the Materials and Methods. Individual data points are included on figures, in addition to the mean value wherever relevant.

(For large datasets, or papers with a very large number of statistical tests, you may upload a single table file with tests, Ns, etc., with reference to sections in the manuscript.)

Group allocation

- Indicate how samples were allocated into experimental groups (in the case of clinical studies, please specify allocation to treatment method); if randomization was used, please also state if restricted randomization was applied
- Indicate if masking was used during group allocation, data collection and/or data analysis

Please outline where this information can be found within the submission (e.g., sections or figure legends), or explain why this information doesn't apply to your submission:

Comparable keratinocyte cultures were either allocated into groups based on genotypes or randomly assigned into treatment groups (for experiments in the *Piezo1*-cKO or PIEZO1-tdTomato background). Quantification was performed using analysis pipelines applied to all conditions equally. Blinding was performed during data analysis where appropriate (Flika analysis, single cell migration analyses etc.). During automated analyses, thresholds were chosen based on objective properties and applied across the board.

Additional data files ("source data")

- We encourage you to upload relevant additional data files, such as numerical data that are represented as a graph in a figure, or as a summary table
- Where provided, these should be in the most useful format, and they can be uploaded as "Source data" files linked to a main figure or table
- Include model definition files including the full list of parameters used
- Include code used for data analysis (e.g., R, MatLab)
- Avoid stating that data files are "available upon request"

Please indicate the figures or tables for which source data files have been provided:

Custom data analysis code for analyzing data shown in Fig. 5D has been made uploaded as a Source Code file; all other code or software utilized is publicly available and has been cited in the Methods section. The datasets plotted in Figure 1E, H, J, L, 2B-E, 3D-G, 4G, 5E, Figure 1-figure supplements 2-3, Figure 2-figure supplements 1-3 and Figure 5-figure supplements 1C and 2 have been uploaded as source data files. Source data files for Figure 5C and 5D have been uploaded to Dryad (https://datadryad.org/stash/share/P3SkFE1Nxxs197lcy4HRpY4SG12-DXEoGgPaUeDdaqc).
